# Therapeutic Approaches Targeting Inflammation in Cardiovascular Disorders

**DOI:** 10.3390/biology7040049

**Published:** 2018-11-16

**Authors:** Daniel P. Jones, Jyoti Patel

**Affiliations:** 1 Bristol Medical School, University of Bristol, Canynge Hall, 39 Whatley Road, Bristol BS8 2PS, UK; bq18532@bristol.ac.uk; 2 Division of Cardiovascular Medicine, Radcliffe Department of Medicine, British Heart Foundation Centre of Research Excellence, University of Oxford, John Radcliffe Hospital, Oxford OX3 9DU, UK; 3 Wellcome Trust Centre for Human Genetics, Radcliffe Department of Medicine, University of Oxford, Oxford OX3 7BN, UK

**Keywords:** inflammation, atherosclerosis, myocardial infarction, heart failure, randomised controlled trial

## Abstract

Cardiovascular disease is a leading cause of morbidity and mortality in the Western world and represents an enormous global health burden. Significant advances have been made in the conservative, medical and surgical management across the range of cardiovascular diseases however the inflammatory components of these diseases have traditionally been neglected. Inflammation is certainly a key component of atherosclerosis, a chronic inflammatory condition, but it is at least correlative and predictive of risk in many other aspects of cardiovascular medicine ranging from heart failure to outcomes following reperfusion strategies. Inflammation therefore represents significant potential for future risk stratification of patients as well as offering new therapeutic targets across cardiovascular medicine. This review explores the role of inflammation in several of the major aspects of cardiovascular medicine focusing on current and possible future examples of the targeting of inflammation in prognosis and therapy. It concludes that future directions of cardiovascular research and clinical practice should seek to identify cohorts of patients with a significant inflammatory component to their cardiovascular condition or reaction to cardiovascular intervention. These patients might benefit from therapeutic strategies mounted against the inflammatory components implicated in their condition.

## 1. Introduction

Cardiovascular medicine has been a notable success of the twentieth century with the development of a variety of effective therapies [1]. In the case of atherosclerosis, for example, management has been transformed by the discovery of a range of modifiable lifestyle factors and novel revascularisation techniques such as coronary artery bypass grafting (CABG) and percutaneous coronary intervention (PCI). However, it was only in the late 20th century that the inflammatory components of these diseases began to be explored [2]. This soon led to the widespread acceptance of the Ross hypothesis [3], which firmly establishes atherosclerosis as a chronic inflammatory disease as opposed to simply a disease of lipid dysfunction.

Further to this, we now have a growing volume of experimental evidence that inflammation has roles in heart failure, myocardial infarction (MI) and responses to cardiac interventions [4,5,6]. Inflammatory biomarkers have now been investigated for correlation and predictive capabilities in many aspects of cardiovascular medicine [7,8] and there have been numerous anti-inflammatory strategies attempted in many of these conditions [9,10]. However, in clinical practice most of these strategies have enjoyed little success with a few notable exceptions (see Table 1, Table 2, Table 3 and Table 4). This is due to a traditionally blunted approach incorporating non-specific inflammatory biomarkers and anti-inflammatory therapies [11].

This review will explore the role of inflammation in the clinical management of atherosclerosis and myocardial infarction; as well as that of heart failure which is a major consequence of these conditions. It will also address the impact of inflammation in the reperfusion strategies used in these conditions, namely CABG and PCI. Finally, it will suggest possible future directions in each of these topics. In future, we envisage an approach that risk stratifies patients according to inflammatory biomarkers and utilises new therapies specifically targeting inflammatory processes unique to cardiovascular disease.

## 2. Atherosclerosis

### 2.1. The use of Inflammatory Biomarkers in Atherosclerosis

Atherosclerosis is a chronic inflammatory condition affecting the medium- and large-sized arteries, characterised by the progressive development of lesions consisting of lipid, fibrosis, and inflammatory cell infiltrate within the tunica media [12]. The progression of these lesions into complex atherosclerotic plaques is in turn associated with vessel stenosis and plaque rupture with the generation of atheromatous thromboembolism [13]. It is the major pathological process underlying several cardiovascular diseases, such as MI and stroke, and is thus a leading contributor to morbidity and mortality [14].

Atherosclerosis has seen intense research activity targeting reductions in C-reactive protein (CRP) as a result of retrospective analysis of primary prevention trials with statins which found that the benefits of statins were not solely due to their reduction of low-density lipoprotein (LDL) [15,16]. Instead, part of the efficacy of statins has been attributed to the suggested anti-inflammatory properties of statins. In most of these trials CRP was shown to predict future cardiovascular events and a CRP level >2 mg/dL predicted a treatment response independent of LDL targets being achieved.

There is now a proposed ‘residual inflammation’ in certain individuals that accounts for cardiovascular risk in the presence of normal or low LDL. Notably, the JUPITER trial (Justification for the Use of Statins in Prevention: an Intervention Trial Evaluating Rosuvastatin) specifically demonstrated that at-risk individuals with elevated high-sensitivity CRP (hs-CRP) but normal cholesterol can significantly reduce their risk of MI and stroke by taking statins [17]. More recently, it has been established that the cardiovascular event hazard ratio of CRP is comparable to that of total cholesterol. These results, in combination with widespread and cheap availability of CRP measurement technology, have seen CRP reduction become the focus of several phase II trials investigating therapies targeting inflammation in cardiovascular disease [8]. However, the use of CRP as a direct marker of cardiovascular inflammation has proved controversial. In stable acute coronary syndrome (ACS) patients for instance, CRP was shown to not be predictive of future cardiovascular events [18]. More compellingly, in phase II trials incorporating imaging modalities of atherosclerotic lesions it has been shown that CRP reduction does not correlate with changes in plaque size or composition [19].

Other pro-inflammatory substances have also been explored: IL-6 administration exacerbates atherosclerosis in mice and it has been shown to be elevated in those with stable cardiovascular disease and is an independent predictor of cardiovascular events [20]. Fibrinogen, an acute phase reactant involved in thrombus formation, has also been investigated and shown to independently predict cardiovascular events [21]. There has also been interest in monocyte phenotypes as an inflammatory biomarker in atherosclerosis. In humans, there is a pro-inflammatory CD14^++^ CD16^+^ monocyte population [22], this population appears to be an intermediate to the murine CX3CR1^lo^/Ly6C^hi^ monocyte population previously discussed [17]. It is expanded relative to the anti-inflammatory population in coronary heart disease and its level correlates with plaque vulnerability as demonstrated by coronary CT studies [23,24]. The level of this population has now been shown in several population studies to be an independent risk factor for future cardiovascular events [25,26]. Even in asymptomatic patients, the level has been shown to be predictive of subclinical atherosclerosis [27].

### 2.2. The Use of Anti-Inflammatory Therapies in Atherosclerosis

The chronic nature of atherosclerosis coupled with the sheer proportion of the population who have a subclinical form makes potent anti-inflammatory therapies with numerous side effects, corticosteroids for example, undesirable. Therefore, the challenge has been to develop specific immunosuppressive or immunomodulatory therapies with minimal side effects. Given the role of oxidized LDL in promoting macrophage activation and inflammation within atherosclerotic lesions [15], specific therapies have been developed to target it, either directly or through anti-oxidant action. The phase III ARISE trial (Aggressive Reduction in Inflammation Stops Events) saw succinobucol, an anti-oxidant and anti-inflammatory, used in the prevention of cardiovascular events following recent ischaemia after promising phase II results. However, it failed to demonstrate efficacy during follow up and instead produced significant elevations in LDL [28].

Phospholipase 2 (PLA2) inhibitors similarly reduce levels of oxidized LDL and so were speculated to beneficial in atherosclerosis. These agents displayed a capacity to reduce inflammatory biomarkers in phase II trials [29] however the STABILITY trial (Stabilisation of atherosclerotic plaque by initiation of darapladib therapy) showed darapladib, another PLA2 inhibitor, failed to reduce cardiovascular mortality, MI or stroke rates in patients with stable heart disease [30]. A different route being attempted focuses on CRP and the cytokine pathways directly. Indeed, therapies targeting tumour necrosis factor (TNF) and IL-6 are already routinely used as disease modifying anti-rheumatic drugs (DMARDs) where they have been shown to protect against acute cardiovascular events [31]. Phase IV trials are now underway to assess the cardioprotective role of these drugs [32].

Most recently, the phase III CANTOS study (Canakinumab ANti-inflammatory Thrombosis Outcome Study) assessed the use of canakinumab (an IL-β targeted monoclonal antibody) in post-MI patients who have a CRP >3 mg/L at the time of recruitment [33]. The results were mixed as whilst the trial managed to significantly reduce cardiovascular events, it did not reduce overall mortality because a reduction in cardiovascular mortality was offset by an increase in deaths due to infection. Finally, given the key role of chemokines in atherosclerosis, chemokine targets might offer potential therapeutic benefit in future. Certainly, in experimental work this approach has produced dramatic effects as demonstrated by the plaque reductions in the ApoE^−/−^ CCR2^−/−^ and ApoE^−/−^ CCR5^−/−^ murine models [34,35] or the anti-atherogenic effects of chemokine inhibitors in in-vivo murine models [36,37]. Whilst long term chemokine inhibition is undesirable, this approach could show efficacy in acute secondary prevention in those with recent cardiovascular events who are noted to have ongoing cardiovascular inflammation. Clinical trials of anti-inflammatory agents in atherosclerosis have been summarised in Table 1.

## 3. Myocardial Infarction

### 3.1. The Use of Inflammatory Biomarkers in Myocardial Infarction

MI describes the death of cardiac tissue due to prolonged ischaemia (>20 min) through the occlusion of a coronary vessel, usually by lumen-reducing atherosclerotic plaques and subsequent thrombus formation [38]. It is one of the leading causes of death in the Western world with over 69,000 deaths in the United Kingdom in 2014 [39]. The inflammatory insult in MI is apparent and thus inflammatory biomarkers have attracted more interest than that seen in subclinical atherosclerosis. The degree of insult seen also suggests that even crude measurements of inflammation such as CRP correlate to the underlying inflammation in the infarction [40]. Consequently, it is now well established that the acute phase reactants hs-CRP, serum amyloid A (SAA) and pregnancy-associated plasma protein (PAPP-A) all positively correlate with traditional cardiac biomarkers such as troponin I or creatinine phosphokinase following acute MI [7,41]. Further to this, these inflammatory biomarkers have all been shown to be able to strongly predict the future risk of coronary events, heart failure and mortality after acute myocardial infarction [42]. Patients with elevated hs-CRP also seem to particularly benefit from statins and anti-platelet agents following acute MI [23].

Similarly, IL-6 and TNFα have predictive value for mortality and future coronary events following acute myocardial infarction [43]. In the FRISC-II trial (FRagmin and Fast Revascularisation during InStability in Coronary artery disease), IL-6 was shown to predict mortality in those presenting with mortality and those with elevated IL-6 were shown to exclusively have a mortality benefit from an early invasive strategy whereas those with normal levels did not. More recently, studies have been published that demonstrate correlation of macrophage migration inhibitory factor (MIF) with infarct size and post-infarct left ventricular ejection fractions suggesting prognostic value with this biomarker as well [44]. Beyond acute phase reactants, it has been shown that neutrophilia is a strong predictor of mortality following acute MI [45]. There is also an expansion of the CD14^++^ CD16^+^ monocyte population (previously discussed) after infarction [46] which has now also been shown to be able to predict future risk of cardiac events [29]. Based on murine models, it is highly likely that this cell type has a role in the inflammatory components of infarction resolution [16].

### 3.2. The Use of Anti-Inflammatory Therapies in Myocardial Infarction

The role of immune cells in scar formation and resolution in MI makes the development of any anti-inflammatory therapies difficult. Such therapy would have to carefully modulate the immune system to prevent excess inflammation whilst allowing wound healing. Nevertheless, several therapies have been tried in this field. Some of the most potent anti-inflammatory agents available are glucocorticoids and, despite concerns, they have been used in several clinical trials of patients following acute MI. A recent meta-analysis demonstrated a 26% relative risk reduction in mortality although this effect was not seen when analysis only included larger studies [47]. Conversely, there was no excessive risk of rupture, easing concerns over poor wound healing. Nevertheless, steroids are associated with volume retention, oedema and hyperglycaemia and so are currently not recommended to be used in the management of acute MI [48]. Another widely used class of anti-inflammatory drugs investigated in the context of acute MI are non-steroidal anti-inflammatory drugs (NSAIDs). However, the evidence consistently shows worse clinical outcome with their use [49]. To refine this therapy, the NUT-2 trial (Non-steroidal anti-inflammatory drugs in Unstable angina Treatment) utilised meloxicam, a cyclo-oxygenase 2 (COX-2) selective inhibitor, which produced significant reductions in mortality and further MI rates after 90 days [50]. Despite this promising early result, a 2006 meta-analysis concluded that selective COX-2 inhibitors are associated with an increased risk of vascular events [51].

The complement cascade has also been investigated as a potential target given its early activation following myocardial infarction and several agents have shown promising results in preclinical studies. Pexelizumab, a monoclonal antibody against C5, demonstrated a significant decrease in mortality in a phase II clinical trial [52] however the APEX-AMI study (Assessment of Pexelizumab in Acute Myocardial Infarction) failed to demonstrate any effect of the agent on mortality after acute MI [53]. Finally, the cytokine system has also been targeted: IL-1 is a pro-inflammatory cytokine and experimental models suggest Il-1 blockade to be beneficial in the context of MI [54]. Recently, two phase II studies utilised anakinra (a recombinant IL-1 receptor agonist) in patients following primary PCI and demonstrated reductions in CRP levels coupled with lower incidence of heart failure at 3 months [55,56]. A larger phase II trial is due to publish its results in late 2018 [57]. Clinical trials of anti-inflammatory agents in MI have been summarised in Table 2.

## 4. Reperfusion Strategies

### Inflammation and Anti-Inflammatory Strategies in Reperfusion Techniques

In patients who have suffered with clinical atherosclerosis and MI, reperfusion is a frequently used therapy [56]. In those identified to be benefit from reperfusion, there are two main treatment modalities: CABG and PCI. In the United Kingdom alone there were 16,791 CABG operations and 92,445 PCI procedures in 2012 [44]. Both procedures are invasive, directly involve manipulation of the coronary vasculature, and necessarily lead to reperfusion of ischaemic areas. Consequently, systemic inflammation is seen following either procedure in the form of elevated cytokine levels [58,59].

This is particularly the case with CABG which not only has the systemic inflammatory response associated with major surgery but the added inflammatory stimulus of cardio-pulmonary bypass (CPB) in on-pump surgery. CPB now has a well-documented inflammatory response through a variety of proposed mechanisms [60]. Indeed, it is plausible that a significant portion of the clear benefits of off-pump surgery are due to a suppressed inflammatory response in this form of CABG [61]. The adverse outcomes associated with a marked inflammatory response following CABG, from post-operative fever to profound organ dysfunction [60,61], have led to concerted attempts to suppress the post-operative inflammatory response. For example, post-operative atrial fibrillation (the most common major complication of CABG) has a well-defined positive correlation with IL-6 and CRP levels post-operatively [62].

Several RCTs have focused on improving the extra-corporeal circuits and whilst they show clinical benefit, it is not however consistently linked to a correlated reduction in inflammation [63]. There have been multiple RCTs utilising corticosteroids, however a Cochrane review on corticosteroids in cardiac surgery showed no benefit to morbidity or mortality [64]. Rosuvastatin has also been incorporated in a recent RCT (Statin Therapy In Cardiac Surgery) where it was administered perioperatively in elective cardiac surgery [65]. Whilst it reduced CRP levels post-operatively, it did not reduce post-operative AF or cardiac damage compared to placebo.

In addition, there have also been several pilot studies utilising a variety of anti-inflammatory strategies in cardiac surgery including delivery of Nitrous Oxide [66] and neutrophil elastase inhibitors [67]. Whilst they showed a reduction in inflammation, clinical outcomes were not measured. In the case of PCI, whilst there is an inflammatory response, the correlation between the degree of inflammation (as measured by typical cytokines) and adverse outcomes has only been reproducible in studies with bare metal stents, not modern stents, although there are new promising biomarkers [68]. Nevertheless, restenosis following PCI is considered an inflammatory response to vascular injury as are other post-PCI complications [69] and thus anti-inflammatory strategies have been attempted. The stent is an obvious point for intervention and the superiority of drug eluting stents (DESs) is now well established [70]. The agents most commonly used are selected for anti-proliferative qualities, such as the rapamycin derivatives, though these agents also display anti-inflammatory activity [71]. A novel agent is titanium–nitric oxide which works exclusively on an anti-oxidative/anti-inflammatory basis [72]; it has so far proved comparable to several DESs. Finally, systemic anti-inflammatory therapy has also been attempted in the form of corticosteroids: The IMPRESS trial (Efficacy of Intramuscular Methyl-prednisone to prevent Restenosis after coronary artery stenting with bare-metal stainless steel stents) demonstrated higher event-free survival following use of prednisone after PCI with bare metal stents (BMS) for instance [73]. The four-year follow up in the CEREA-DES trial (Cortisone plus BMS or DES versus BMS alone to eliminate restenosis) showed these positive effects were maintained and that this combination is comparable to drug eluting stents [74]. Clinical trials of anti-inflammatory agents in PCI/CABG have been summarised in Table 3.

## 5. Heart Failure

### 5.1. Inflammation in Heart Failure

One of the most serious consequences of atherosclerosis and MI, particularly in the absence or failure of reperfusion, is the development of heart failure. Heart failure is defined as inadequate cardiac function and affects 1–2% of the adult population. It is associated with a poor prognosis with 1-year mortality at 30–40% [44]. Since 1990, there has been a recognised contribution of inflammation to heart failure following a study by Levine et al. which demonstrated elevated levels of TNF in heart failure patients [75]. Subsequently, TNF has been demonstrated, in animal studies, to have profound negative inotropic effects whether that be via direct injection [76] or through cardiac-restricted gene overexpression [77].

Long term animal studies have also been able to demonstrate the capacity of TNF to induce deleterious left ventricular remodelling [78]. Indeed, experimental models have now linked several aspects of the innate immune system to myocyte apoptosis, myocardial fibrosis, foetal cardiac gene expression and myocardial matrix metalloproteinase activation [79]. Analogous to the neurohormonal pathways implicated in heart failure, inflammation in heart failure appears to initially be an attempt to restore homeostasis but in the absence of recovery chronically contributes to the disease process. It is however difficult to modulate the immune system in heart failure in such a way as to enable attempts to restore homeostasis whilst sparing the heart from the chronic deleterious effects of uncontrolled inflammation.

### 5.2. The Use of Inflammatory Biomarkers in Heart Failure

There is a clear and established role for the use of inflammatory biomarkers in heart failure. CRP has been shown to elevated in more severe grades of heart failure and can independently predict morbidity and mortality in heart failure [80] and TNF levels have been shown to do the same [81]. Further to this, the Food and Drugs Administration (FDA) has recently approved two prognostic inflammatory biomarkers in heart failure, these being soluble ST2 (a receptor for IL-33) and galectin-3 (a lectin released by activated macrophages and damaged cells). There is now also interest in pentraxin-3 (a novel cytokine) as a biomarker in heart failure too [82].

In similarity to subclinical atherosclerosis and MI, there is now also an implicated role for the CD14^++^ CD16^+^ monocyte population. Recently, this population was shown to be significantly increased in patients with congestive heart failure and the level of this population correlates to the severity of heart failure, left ventricular ejection fraction (LVEF) and pro-brain natriuretic peptide levels [83]. This population may well be directly contributing to heart failure and the potential for it as an inflammatory biomarker in this condition is clear.

### 5.3. The Use of Anti-Inflammatory Therapies in Heart Failure

With the apparent contribution of TNF to heart failure pathophysiology, it has emerged as a distinct target in clinical trials. Pentoxifylline and thalidomide have both been shown to reduce TNF production as well as other pro-inflammatory mediators and in several small clinical trials have demonstrated a capacity to improve LVEF and symptoms in patients with heart failure [84,85] though not mortality.

Further to this, etanercept has also been trialled in the RECOVER (Research into Etanercept CytOkine Antagonism in VentriculaR dysfunction) and RENAISSANCE (Randomised Etanercept North American Strategy to Study Antagonism of CytokinEs) studies (collectively analysed as the Randomised EtaNErcept Worldwide evALuation) with a combined total of 1500 heart failure patients. These studies, however, failed to show a benefit of etanercept on a variety of primary outcomes and the RENAISSANCE trial specifically demonstrated a significant worsening of heart failure in patients [86]. Infliximab (a monoclonal antibody against TNF) was also trialled in the ATTACH study (Anti-TNF Therapy Against Congestive Herat failure) and increased mortality and hospitalization rates at higher doses [87].

Dexamethasone has also been trialled in idiopathic dilated cardiomyopathy patients with heart failure and was able to demonstrate no significant benefits over placebo [88]. Statins have been the subject of interest in heart failure as well. The CORONA [89] (Crestor versus placebo in subjects with Heart Failure) and GISSI-HF [90] (Effect of rosuvastatin in patients with chronic heart failure) trials were both large RCTs that sought to investigate the use of rosuvastatin as an intervention in heart failure. Neither could demonstrate an effect of rosuvastatin on death or hospital admission due to cardiovascular reasons. However, post hoc analysis of the CORONA trial did reveal benefits to patients with baseline CRP levels >2 mg/dL in line with the results we saw in subclinical atherosclerosis [91].

Finally, methotrexate has also been trialled in heart failure patients due to its ability to reduce heart failure rates in patients with rheumatoid arthritis [35]. The METIS trial (Methotrexate therapy on the physical capacity of patients with Ischaemic heart failure) was a small clinical trial that evaluated methotrexate against a placebo in 50 heart failure patients, but it found no significant differences [92]. However, heart failure related outcomes following methotrexate treatment are being included in the much larger CIRT study which should have greater power to detect benefits [93]. Clinical trials of anti-inflammatory agents in heart failure have been summarised in Table 4.

## 6. Conclusions

In conclusion, there is a prominent role for inflammation in several aspects of cardiovascular medicine from atherosclerosis and one of its major consequences in MI. Inflammation is involved in the reperfusion strategies used to address these conditions as well as heart failure, one of the major consequences of MI and failed reperfusion.

This review demonstrates groups of patients who have a significant inflammatory component contributing to their cardiovascular disease. These patients can often be detected with the use of current inflammatory biomarkers with several more promising and robust biomarkers on the horizon. This stratification has the potential to improve prognostic accuracy and identify distinct patient groups who might benefit from anti-inflammatory strategies. There is evidence already that such strategies are of benefit in cardiovascular medicine and there exists the potential to expand both the scope and efficacy of such strategies.

In future, we will hopefully be able to identify ever more specific targets to the inflammatory component of a patient’s cardiovascular disease which in turn will allow specifically tailored therapies that may prove to be of benefit. This is in stark contrast to the blunt anti-inflammatory therapies discussed in this review. With all of this in mind, the links between basic, translational and clinical research will prove more important than ever. Indeed, this review already demonstrates how quickly these therapeutics have gone from the bench side to clinic. If we are to have success, then the relevant research scientists will need to closely follow these clinical trials in order to refine their hypotheses. In turn, clinical scientists will need to keep abreast of developments in translational research in order to identify and trial promising therapeutics sooner rather than later.

## Figures and Tables

**Table 1:** Notable clinical trials of anti-inflammatory agents in atherosclerosis. CRP: C-reactive protein; PLA2: phospholipase 2; MI: myocardial infarction.

Trial	Intervention	Design	Outcome
ARISE	Succinobucol in those with previous acute coronary syndrome	Double-blinded randomised controlled trial (RCT) with placebo as control; *n* = 6144	No significant clinical benefits over placebo
JUPITER	Rosuvastatin in those without hyperlipidaemia but with elevated CRP	Double blinded RCT with placebo as control; *n* = 17,802	Rosuvasatin significantly reduced rates of stroke, MI or cardiovascular death
STABILITY	Darapladib (PLA2 inhibitor) in stable coronary heart disease	Double blinded RCT with placebo as control; *n* = 15,828	Darapladib did not significantly affect rates of MI, stroke, or cardiovascular death
CANTOS	Canakinumab (Il-1 inhibitor) in those with previous MI and raised baseline CRP	Double blinded RCT with different dose groups and placebo as control; *n* = 10,061	Canakinumab doses of 150 mg or more reduced rates of MI but not overall mortality

**Table 2:** Notable clinical trials and meta-analyses of anti-inflammatory agents in MI. ACS: acute coronary syndrome; PCI: percutaneous coronary intervention.

Trial/Meta-Analysis	Intervention	Design	Outcome
NUT-2	Meloxicam (COX2 inhibitor) plus standard treatment in ACS patients	Single blinded RCT with standard treatment as control; *n* = 120	Significant reduction in recurrent MI and deaths with meloxicam
APEX-AMI	Pexelizumab (anti-C5) in patients receiving PCI for MI	Double blinded RCT with placebo as control; *n* = 5745	No significant differences between treatment or placebo
FRISC-II	Early invasive strategy post-MI for those with significant risk factors including IL-6/CRP levels	Risk stratification into intervention or normal treatment. Raised IL-6/CRP levels as risk factors; *n* = 2457	Early invasive strategy in these patients significantly reduced rates of MI but not mortality
Pooled results of VCU-ART1 and VCU-ART2	Anakinra (IL-1 antagonist) for post MI patients	Double blinded RCTs with placebo as control; combined *n* = 70	Significant reduction in developing heart failure post MI with anakinra
Meta-analysis of corticosteroid treatment in MI	Glucocorticoids in post-MI patients	Meta-analysis of 11 controlled studies of glucocorticoids versus placebo	No significant clinical benefits with glucocorticoids
Meta-analysis of COX-2 inhibitor use	COX-2 inhibitors in a variety of patient populations	Met-analysis of 138 RCTs of COX-2 inhibitors versus placebo/NSAID/both	COX-2 inhibitors significantly increase risk of MI

**Table 3:** Notable clinical studies and meta-analyses of anti-inflammatory therapies in PCI/ coronary artery bypass grafting (CABG).

Trial/Meta-Analysis	Intervention	Design	Outcome
STICS	Rosuvastatin in elective cardiac surgery	Double blinded RCT with a placebo as control; *n* = 1922	Rosuvastatin did reduce CRP but did not significantly affect post-operative outcomes
CEREA-DES	Prednisone with bare metal stents in PCI	Single blinded RCT with drug eluting stents and bare metal stents without prednisone as other treatments; *n* = 375	Bare metal stents with prednisone and drug eluting stents both have higher event free survival compared to bare metal stents only
Meta-analysis of corticosteroids in cardiac surgery	High dose prophylactic steroids administered in on-pump CABG	54 RCTS included of variable quality; total *n* = 3615	No significant clinical benefits with corticosteroids

**Table 4:** Notable clinical studies of anti-inflammatory therapies in heart failure patients.

Trial	Intervention	Design	Outcome
RENEWAL	Etanercept in heart failure patients (NYHA II-IV)	Two double-blinded RCTs with placebo as control; *n* = 2048	No significant clinical benefits of etanercept over placebo
ATTACH	Infliximab in heart failure patients (NYHA III-IV)	Double-blinded RCT with placebo as control; *n* = 150	No significant clinical benefits. High dose infliximab increased mortality
Prednisone in Idiopathic Dilated Cardiomyotpathy	Prednisone in patients with idiopathic dilated cardiomyopathy	Single-blinded RCT with placebo as control; *n* = 102	No significant clinical benefits with prednisone over placebo
CORONA	Rosuvastatin in heart failure patients (NYHA II-IV)	Double-blinded RCT with placebo as control; *n* = 5011	Reduction in hospitalization rates if patient has multiple admissions or CRP >2
GISSI-HF	Rosuvastatin in heart failure patients (NYHA II-IV)	Double-blinded RCT with placebo as control; *n* = 4574	No significant clinical benefits with rosuvastatin over placebo
METIS	Methotrexate plus folic acid in ischaemic heart failure patients	Double-blinded RCT with placebo and folic acid as control; *n* = 50	No significant clinical benefits with methotrexate over placebo
